# Diabetes as a potential compounding factor in COVID-19-mediated male subfertility

**DOI:** 10.1186/s13578-022-00766-x

**Published:** 2022-03-20

**Authors:** Qingkui Jiang, Thomas Linn, Karl Drlica, Lanbo Shi

**Affiliations:** 1 Public Health Research Institute, New Jersey Medical School, Rutgers Biomedical and Health Sciences, Rutgers The State University of New Jersey, Newark, NJ USA; 2 Clinical Research Unit, Centre of Internal Medicine, Justus-Liebig-University (JLU), Giessen, Germany; 3 Public Health Research Institute and Department of Microbiology, Biochemistry, and Molecular Genetics, New Jersey Medical School, Rutgers Biomedical and Health Sciences, Rutgers The State University of New Jersey, Newark, NJ USA

**Keywords:** SARS-CoV-2, Inflammation, Diabetes, Spermatogenesis, Male reproductive hormones, Male subfertility

## Abstract

Recent work indicates that male fertility is compromised by SARS-CoV-2 infection. Direct effects derive from the presence of viral entry receptors (ACE2 and/or CD147) on the surface of testicular cells, such as spermatocytes, Sertoli cells, and Leydig cells. Indirect effects on testis and concentrations of male reproductive hormones derive from (1) virus-stimulated inflammation; (2) viral-induced diabetes, and (3) an interaction between diabetes and inflammation that exacerbates the deleterious effect of each perturbation. Reproductive hormones affected include testosterone, luteinizing hormone, and follicle-stimulating hormone. Reduction of male fertility is also observed with other viral infections, but the global pandemic of COVID-19 makes demographic and public health implications of reduced male fertility of major concern, especially if it occurs in the absence of serious symptoms that would otherwise encourage vaccination. Clinical documentation of COVID-19-associated male subfertility is now warranted to obtain quantitative relationships between infection severity and subfertility; mechanistic studies using animal models may reveal ways to mitigate the problem. In the meantime, the possibility of subfertility due to COVID-19 should enter considerations of vaccine hesitancy by reproductive-age males.

## Introduction

Although most persons infected with SARS-CoV-2 experience only mild-to-moderate respiratory illness and recover without special treatment, some progress to severe disease that is often associated with acute respiratory distress syndrome [1]. Thus, COVID-19 was initially considered to be a lung disease [2]. Subsequent experimental and clinical studies now indicate that SARS-CoV-2 infection damages multiple organs, including brain [3], kidney [4], pancreas [5], and testis [6]. Moreover, some infected individuals suffer from long-term sequelae, such as extreme fatigue, muscle weakness, low-grade fever, new onset of diabetes, and hypertension [7, 8]. Therefore, COVID-19 cannot be treated as a common viral infection that appears to come and go.

Key features of the male reproductive system make it vulnerable to damage by COVID-19. For example, spermatogonia, Sertoli cells, and Leydig cells reside outside the blood-testis barrier and are therefore susceptible to viral infection. Direct SARS-CoV-2 infection of cells associated with sperm development and effects of inflammation are both likely to compromise male reproductive potential [9]. Moreover, synthesis and secretion of male reproductive hormones, such as gonadotropin-releasing hormone (GnRH), follicle-stimulating hormone (FSH), luteinizing hormone (LH), and testosterone are sensitive to various types of inflammation [10]. These observations, plus several other lines of evidence, indicate a negative impact of SARS-CoV-2 infection on male fertility, at least during serious diseases [6].

The mechanisms underlying testicular dysfunction in male COVID-19 patients remain poorly understood. In addition to effects from direct infection and inflammation, an under-appreciated factor is diabetes. This well-documented risk factor for male subfertility [11, 12] may contribute significantly to low fertility in COVID-19. Indeed, diabetes often occurs in COVID-19-patients with pre-diabetes and with patients lacking prior glucose metabolism disorders [13]. Below we describe viremia-induced male subfertility, followed by a discussion of potential mechanisms by which SARS-CoV-2 infection causes male hypogonadism. We argue that diabetes aggravates the negative impact of SARS-CoV-2 infection on male reproductive potential.

## Viremia and male subfertility

### Lessons from other viral infections

Viral infections have long been associated with male subfertility. In a recent example, men infected with Zika virus exhibited decreased semen quality and the presence of viral RNA in spermatozoa [14]. Findings from murine infection models associate reduced semen quality with persistence of Zika virus and induction of pro-inflammatory cytokines/chemokines in testicular cells (Sertoli, Leydig, and epididymal epithelial cells) [15, 16]. In another example, 15 to 30% of adult males infected with mumps virus develop epididymo-orchitis and subfertility [17]; murine studies indicate that the underlying mechanism is related to increased innate immune response in Sertoli, Leydig, and germ cells. Similarly, infection of human spermatozoa by HIV compromises male fertility [18, 19], as demonstrated by detectable virus in seminal fluids, severe testicular atrophy, and chronic orchitis with progressive hypogonadism [20–22]. In a fourth example, patients with chronic infection by hepatitis virus C exhibit a decrease in semen volume, sperm count, sperm motility, and testosterone [23]. Finally, a meta-analysis of the human papillomavirus in semen reveals a positive correlation between virus positivity and an elevated risk of male infertility [24]. Thus, a precedence exists for viral infection-induced male subfertility: SARS-CoV-2 infection is simply another example.

### SARS-CoV-2 in testes

**Fig. 1:**
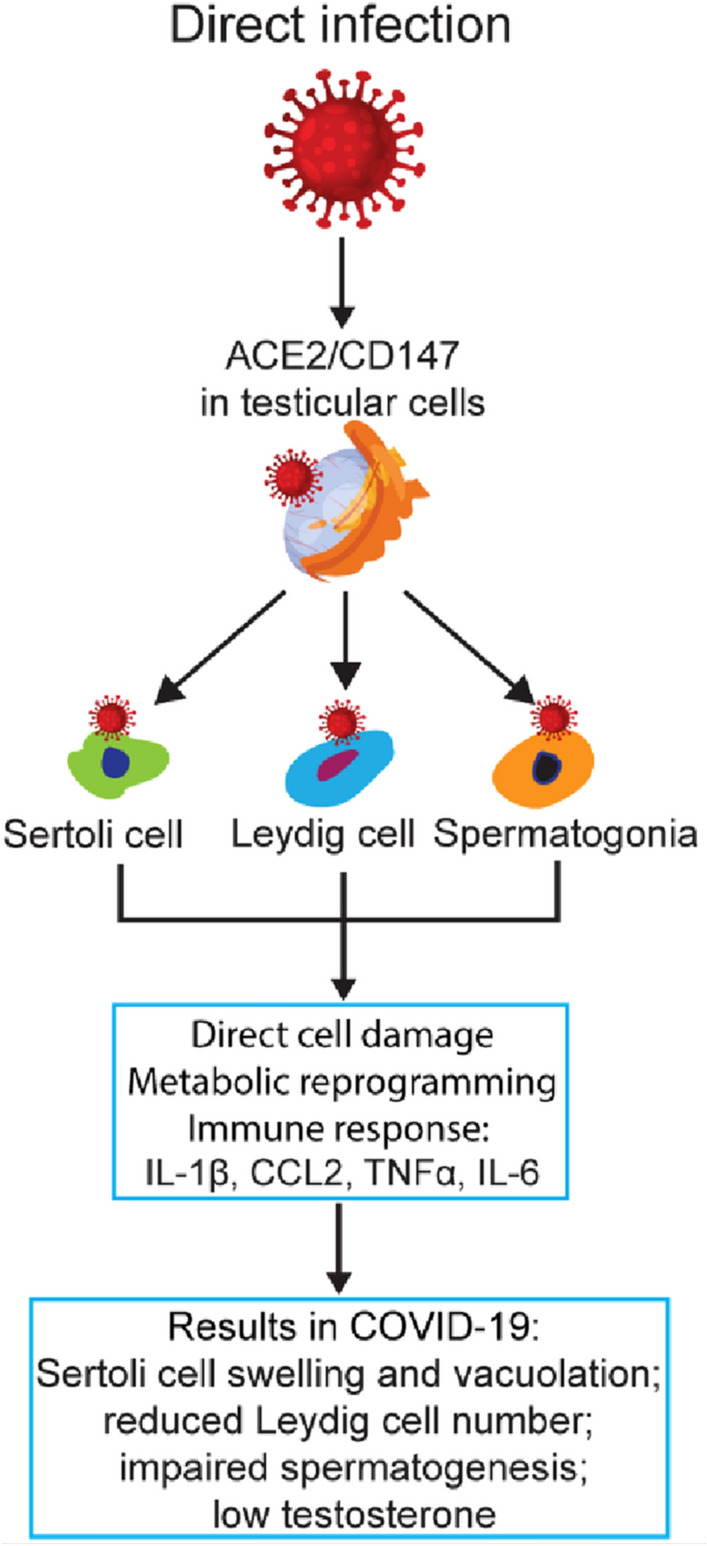
Direct infection of testicular cells by SARS-CoV-2. By binding to the highly expressed viral entry receptors on testicular cells, specifically the Sertoli cells, Leydig cells, and spermatogonia, SARS-CoV-2 may infect these cells and cause direct cell damage, metabolic reprogramming, and/or immune responses. The result would be cell dysfunction and apoptosis

It is well known that SARS-CoV-2 infects host cells through an interaction of its spike protein with the host ACE2 receptor, which is expressed on the surface of several cell types [25]. High-level expression of ACE2 in human testes [26], mainly in spermatogonia, Leydig cells, and Sertoli cells [27], suggests testis susceptibility to circulating virus. In addition, CD147, which mediates host-cell entry for viruses such as HIV [28] and SARS-CoV [29], also functions as a receptor for SARS-CoV-2 [30]. This observation has important implications, given that CD147 is expressed in Sertoli cells, Leydig cells, and germ cells of all stages, and that it is also a critical regulator in spermatogenesis [31]. Thus, SARS-CoV-2 may impact testicular function by specific binding to the two different receptors (Fig. 1).

Patient data are consistent with direct effects of SARS-CoV-2 on male fertility. Two studies of severe disease detected SARS-CoV-2 particles in testicular cells. In one, two out of five postmortem testis biopsies revealed virus [6]; in the other, one out of five were virus-positive postmortem, and one live biopsy was virus-positive [32]. Clinical manifestations of testicular injury in COVID-19 include orchitis [33], structural change of testis [34], spermatogenesis impairment [6, 35], and significant changes of major male reproductive hormones, such as increased serum levels of LH and FSH and decreased testosterone [36, 37]. Since testicular damage is often found without direct evidence of virus in the testis, other factors likely contribute indirectly to testicular dysfunction. Below we discuss two indirect COVID-19-associated factors, inflammation and diabetes; we postulate that their interaction exacerbates male reproductive damage.

### Viral infection-induced inflammation

Inflammation in the reproductive tract is associated with male subfertility. For example, asymptomatic focal inflammatory lesions are found in approximately half of testicular biopsies from males exhibiting impaired fertility [38], and levels of proinflammatory cytokines (IL-1β, IL-6 and TNF) are elevated in seminal plasma of infertile males [39]. Mechanisms of inflammation on testicular dysfunction have been revealed by in vitro and in vivo studies. Inflammatory molecules, either produced locally or derived from systemic inflammation, damage germ cells and somatic cells by activating the Janus kinase-signal transducer and activator of transcription pathway and the MAPK pathway [40, 41]. Our recent work also shows that testicular inflammation is associated with impaired spermatogenesis and Leydig cell dysfunction and apoptosis [42]. Inflammation also acts by dysregulating hormone secretion. For example, in the mouse hypothalamic GnRH neuronal cell line, neuroinflammation-induced cytokines, specifically the leukemia inhibitory factor, repress GnRH gene expression by activating the MAPK pathway [43]. Proinflammatory cytokines, such as IL-1β, TNF and IL-6, also negatively affect pituitary function, reducing the synthesis and/or secretion of FSH and LH [44]. Thus, inflammation appears to contribute to male subfertility at multiple levels.

**Fig. 2:**
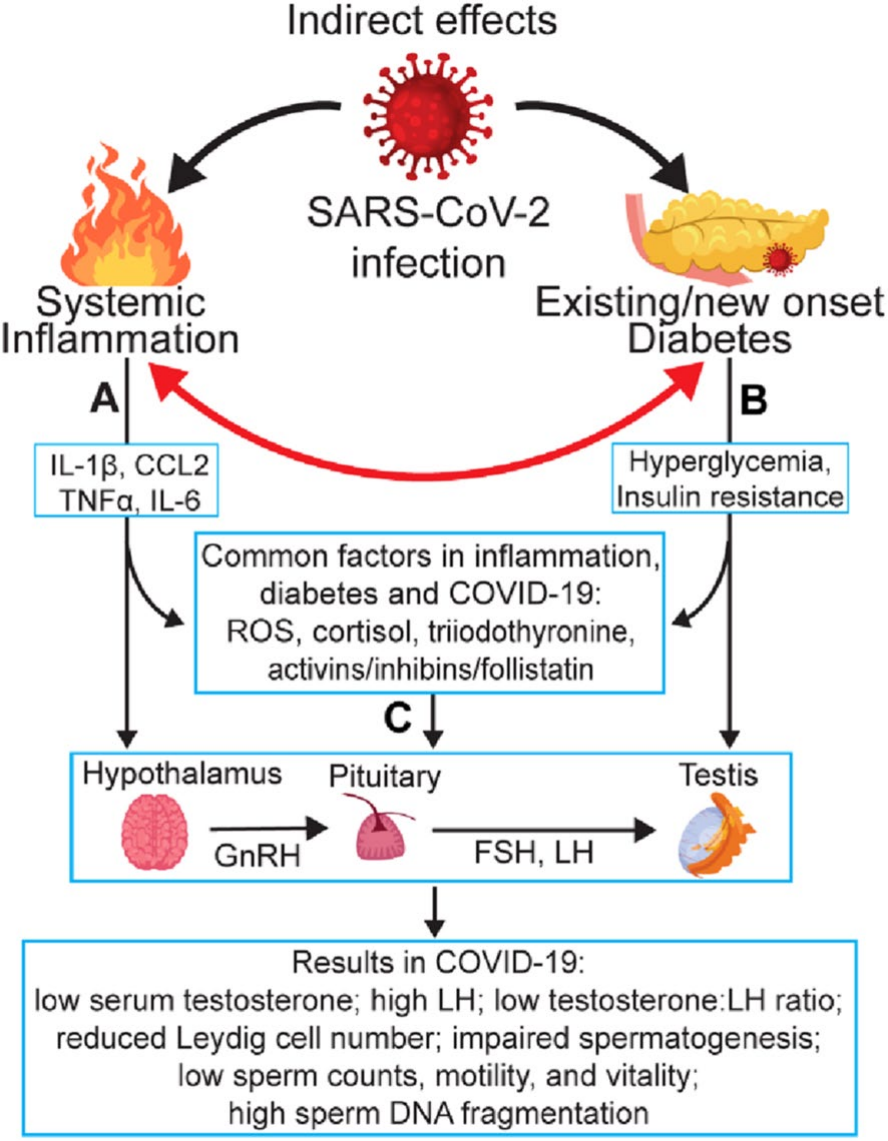
Indirect effects of SARS-CoV-2 infection on male fertility. Three interconnected pathways during COVID-19 negatively impact male fertility. Inflammation (**A**) and diabetes (**B**) each serve as a secondary factor of SARS-CoV-2 infection to independently affect the hypothalamic-pituitary-testis (HPT) axis and/or testicular cells by direct impact or through other mediators. **C** The interaction between inflammation and diabetes exacerbates the deleterious effect of each individual perturbation, leading to male subfertility

COVID-19 is an inflammatory disease, as defined by systemic production and secretion of proinflammatory cytokines/chemokines [45]. Since infection and inflammation are important triggers of male infertility in general [46], inflammation can be a good candidate for poor male reproductive outcome associated with COVID-19 (Fig. 2A). Indeed, seminiferous tubular injury and reduced numbers of Leydig cells associated with mild inflammation are seen in the testis of COVID-19 patients [47]. Moreover, clinical data from COVID-19 patients indicate that reduction of serum testosterone is negatively associated with levels of C-reactive protein (a marker of inflammation) and predictive of poor prognosis for COVID-19 patients [36, 48]. Little else is known about the mechanisms underlying inflammation effects on male reproductive outcome in COVID-19.

Although inflammation recedes in COVID-19 patients when they recover from primary disease, new evidence indicates that the effects of SARS-CoV-2 infection on male fertility are long-lasting. For example, about a month after recovering from COVID-19, four out of five patients admitted to the intensive care unit, three out of 26 hospitalized, and one among 12 non-hospitalized showed azoospermia [49]. A case study also described a male of reproductive age who exhibited reduced sperm concentration and sperm count, increased abnormal sperm morphology, and reduced sperm vitality four months after recovery from COVID-19 [50]. Thus, factors other than the initial inflammation during COVID-19 persist that negatively affect male fertility. Below we consider diabetes as a persisting factor in the reduction of male fertility because hyperglycemia is frequently seen in COVID-19 patients (46% during hospitalization, 36% of which continue to show hyperglycemia in the following six months) [51] and because diabetes is a major risk factor for male subfertility.

## COVID-19-associated diabetes reduces male fertility

### Lessons from other viral infections

Infection with a variety of viruses is often associated with the onset of diabetes. For example, cytomegalovirus infection contributes to the pathogenesis of both type-1 and type-2 diabetes [52, 53]. Indeed, persons with a history of cytomegalovirus infection exhibit a 12-fold greater chance for developing type-2 diabetes [54]. Diabetes is also highly prevalent in hepatitis C virus-infected individuals (50% of adult cirrhotic patients infected with the virus develop diabetes mellitus compared to 9% of patients with cirrhosis in the absence of hepatitis C infection [55]). In an example similar to SARS-COV-2 infection, about 50% of SARS-CoV-infected patients lacking a history of diabetes developed high blood glucose levels within two weeks of hospitalization. Thus, SARS-CoV infection can markedly influence glucose homeostasis, at least in the acute phase of disease [56]. The onset of diabetes during SARS-CoV-2 infection may be common rather than exceptional; if so, diabetes is expected to be a significant contributor to male subfertility.

### Reciprocal relationship between COVID-19 and diabetes

In a case-series study of 5,700 patients hospitalized with COVID-19 in the New York City area, 34% were diabetic [57]. Diabetes was associated with disease progression to severe COVID-19 (odds ratio of 3.07) [58] and with a 3.2-fold increase in COVID-19-related mortality [59]. The insulin resistance marker (triglyceride and glucose index) was also found to associate with the severity and mortality of COVID-19 [60]. Uncontrolled blood glucose levels may contribute to the development of severe disease in diabetic COVID-19 patients by promoting virus replication and cytokine production in monocytes [61]. High glucose levels also result in an exacerbated pro-inflammatory response and acute metabolic decompensation of pre-existing diabetes [62]. These observations clearly show that diabetes is a risk factor for worse prognosis in COVID-19.

A reciprocal relationship also appears to exist. SARS-CoV-2 infection is reported to lead to the onset of insulin resistance and acute hyperglycemia in people without diabetes and to the development of diabetes in patients with pre-diabetes [63–65]. Two underlying mechanisms are likely involved. First, infection of pancreatic cells by SARS-CoV-2 can lead to the onset of hyperglycemia and/or diabetes. Indeed, ACE2 mRNA is expressed in both the islets and exocrine glands of the pancreas at a level about 1.6 times higher than seen in the lung [66]. Moreover, viral infiltration of pancreatic islets [67] and pancreatic injury [66] are seen with COVID-19 patients; such infiltration might contribute to an acute β-cell dysfunction, leading to acute hyperglycemia [56]. Second, proinflammatory cytokines and reactive oxygen species (ROS), produced during SARS-CoV-2 infection, are known to induce insulin resistance [68]. In the case of circulating inflammatory markers in obesity, such as TNF, activation of the IKKβ/NFκB pathway is associated with induction of insulin resistance and type-2 diabetes in mice [69]. In addition, elevated ROS levels trigger insulin resistance in cell culture and mouse models [70]. Thus, poor glycemic control and a cytokine storm during SARS-CoV-2 infection can act as a vicious cycle, reciprocally leading to severe symptoms and poor prognosis of COVID-19 patients with diabetes [71, 72]. An international collaboration, the CoviDIAB Project (https://covidiab.e-dendrite.com), seeks to understand the pathogenesis, management, and outcomes of COVID-19-related diabetes [64]. To this effort we add male subfertility as another potentially serious consequence of COVID-19-associated poor glycemic control (Fig. 2B), as discussed below.

### Diabetes during COVID-19 affects male fertility

We and others have shown that diabetes is associated with testicular damage in murine models [42, 73, 74]. As expected, treatments that lower hyperglycemia can restore spermatogenesis and steroidogenesis in diabetic animals (increased sperm count, sperm motility, and testosterone) [73, 75, 76]. In addition, administration of metformin, the first-line treatment for type-2 diabetes in men with diabetic complications, improves sperm concentration and sperm chromatin integrity [77].

Mechanistic studies using animal models show that diabetes disrupts the function of glucose transporters in spermatozoa and Leydig cells [78]. Hyperglycemia damages testes by activating two major pathways, the diacylglycerol-protein kinase C pathway and the polyol pathway [79]. Overall, these findings indicate that the onset of hyperglycemia, even in the absence of obvious testicular viral infection or inflammation, can lead to poor reproductive outcomes in COVID-19 patients (Fig. 2B) [71, 72].

### Inflammation-diabetes interaction

Diabetes, particularly type-2 diabetes, is widely accepted as an inflammatory disease [80]. In diabetes, hyperglycemia and inflammation appear to stimulate each other. On one hand, hyperglycemia often correlates with elevated systemic inflammation. For example, an acute increase in plasma IL-6, TNF, and IL-18 is observed in humans after receiving intravenous glucose; the increase is more pronounced in subjects having impaired glucose tolerance [81]. Moreover, hyperglycemia drives IL-1β production in obesity through activation of the thioredoxin-interacting protein [82]. On the other hand, inflammation has long been recognized as a critical player in the onset and/or progression of diabetes [83]. It is known that TNF-signaling cascades activate the c-Jun amino-terminal kinase, leading to phosphorylation of serine 307 in insulin receptor substrate-1. This event causes insulin unresponsiveness [84]. Additionally, IL-1β affects β-cells through a direct cytotoxic effect [85] and/or by driving tissue inflammation within islet cells [86], thereby impairing β-cell function and insulin sensitivity. IL-6 also impairs insulin metabolism by increasing the expression and activity of the insulin-degrading enzyme [87]. Finally, pro-inflammatory cytokine-related ROS overproduction, associated with high neutrophil-to-lymphocyte ratio during SARS-CoV-2 infection [68], can trigger insulin resistance that is observed both in vitro and in vivo [70]. Collectively, the interaction of diabetes and inflammation likely increases complications with a variety of diseases, one of which is male subfertility.

### Inflammation-diabetes interaction worsens male subfertility during COVID-19

We recently found that hyperglycemia interacts with inflammation to cause hypogonadism in diabetic and pre-diabetic mice [42], thereby linking diabetes, inflammation, and male subfertility. In this work, we used homozygous, leptin-resistant diabetic *db/db* mice that are characterized by hypogonadism, hyperglycemia, and testicular inflammation with increased IL-1β and chemokine (C–C motif) ligand 2 (CCL2). The latter is a key member of the cytokine storm associated with COVID-19 severity [88]. Chemical inhibition of CCL2 protects murine and human Leydig cells from malfunction and apoptosis, it reverses the diabetic condition, and it ameliorates hypogonadism in the *db/db* mice [42, 89]. Moreover, in a diabetes model induced by a high-fat diet, *Ccl2*-deficient mice are protected from development of diabetes and testicular dysfunction [42].

Overall, findings from Leydig cell work and mouse studies fit with clinical studies in which a reduction of serum levels of CCL2 is associated with improvement of diabetic conditions and recovery from hypogonadism in a cohort of infertile men having a history of metabolic syndrome [42]. Although we did not address the direct impact of diabetes on subfertility in our murine and clinical studies, the observation that hypogonadism reverses with improved glucose control and reduced inflammation argues for a functional role of diabetes in dysregulation of male reproduction.

Thus, it is likely that deterioration of glycemic control associated with SARS-COV-2 infection has a greater negative impact on male fertility in patients with diabetes or prediabetes. Male COVID-19 patients experiencing both a cytokine storm and diabetes could exhibit severely harmed reproductive potential through compounding effects of two aggravating factors on testicular cells. The result is impaired spermatogenesis, Sertoli and Leydig cell dysfunction and loss, and a dysregulated immune environment in testes [42, 74, 79]. Although COVID-19-associated inflammation is transient, diabetes can be a long-lasting complication, emphasizing the long-term threat of COVID-19 to male fertility (Fig. 2C).

Metformin has been shown to result in an overall improvement of male fertility in diabetic animal models [90–92] and patients [77]. Given its nature as a glucose-lowering and anti-inflammatory drug [93], and that both high glucose and excessive inflammation cause male hypogonadism based on our studies [42, 79], we would speculate that metformin could, at least to a certain extent, protect male diabetic patients from COVID-19-induced subfertility. However, various factors, including severity of diabetes, other complications such as obesity, hypertension and hyperlipidaemia, as well as dosage and duration of metformin treatment, which are reported to influence the effectiveness of metformin on male fertility in diabetic subjects [94], could complicate the unique circumstances in COVID-19.

### Inflammation and diabetes share factors contributing to male subfertility

#### Reactive oxygen species

Although low levels of ROS are needed as signaling molecules, overproduction of ROS under inflammatory and/or diabetic conditions often leads to oxidative stress expected to damage cell functions. Several studies indicate that inflammation is accompanied by ROS stress. One study reports that treatment with cytokines (TNF, IL-1β, and IFN-γ) increases ROS in human retinal pigment epithelial cells [95]. In another, glomerular cells treated with TNF and IL-6 show ROS-mediated stress and damaged glomerular permeability [96]. Similar to inflammation, diabetes is also associated with increased ROS stress. Hyperglycemia in diabetic patients elevate ROS production through mitochondrial respiratory chain enzymes, peroxidases, lipoxygenases, nitric oxide synthases, cyclooxygenases, and xanthine oxidases [97]. Increased ROS level has also been observed in pancreatic islets of diabetic mice [98]. Collectively these findings suggest an important role for ROS in inflammation and diabetes-related complications.

In terms of male fertility, low levels of ROS are necessary for sperm capacitation and hyperactivation, but elevated levels of ROS likely contribute to male subfertility. For example, presumptive ROS-mediated sperm defects, such as poor sperm membrane integrity and high levels of DNA fragmentation, are found in 30 to 80% of cases of infertile males [99]. The high content of polyunsaturated fatty acids in germ-cell plasma membranes makes them particularly vulnerable to ROS-mediated damage [100]. Supraphysiological ROS levels also interfere with Sertoli cell function by repressing the expression of integrin beta-1, a key surface molecule for the interaction of Sertoli cells with germ cells, and the expression of connexin-43, a key component of gap junctions [101]. Overall, elevated levels of ROS in COVID-19 patients, which have been implicated in the dysfunction and damage of multiple organs [68, 102], may also have a negative impact on the male reproductive tract.

#### Cortisol

Cortisol is a glucocorticoid secreted by the adrenal glands. Its increase under stressful conditions, such as diabetes or inflammation, provides the body with an energy boost for blood glucose control, inflammation reduction, and maintenance of metabolic homeostasis [103]. As expected, a significant increase of serum cortisol concentration is seen in COVID-19 patients [104]. However, excessive amounts of cortisol release are linked to detrimental effects on male fertility. For example, elevated serum and salivary cortisol levels correlate with higher incidence of male erectile dysfunction [105]. Our studies with mice show high levels of cortisol in testicular interstitial fluid and serum of mice with subfertility [42], further supporting the idea that cortisol can have a negative role in male fertility. We postulate that the combination of inflammation and diabetes in COVID-19 can lead to prolonged excessive levels of cortisol that disrupt optimal hormonal regulation of male fertility; such is found in rats with decreased LH secretion and impaired Leydig cell function [106, 107].

#### Triiodothyronine

This thyroid hormone is a potent regulator of metabolism and development. Low serum levels of triiodothyronine in patients with nonthyroidal illnesses [108] is considered beneficial, as it prevents excessive tissue catabolism [109]. COVID-19 patients have reduced concentrations of triiodothyronine (64–84 ng/dL in COVID-19 vs. 82–117 ng/dL in non-COVID-19 patients with 95% confidence interval; *p* < 0.01) [110]. From a physiological perspective, this decrease is likely caused by SARS-CoV-2-induced inflammation and/or diabetes, as inflammatory cytokines (IL-1, IL-6,and TNF) are known to inhibit the peripheral conversion of thyroxine to triiodothyronine [111]. Moreover, free triiodothyronine is inversely related to the prevalence of both type-1 [112] and type-2 [113] diabetes in euthyroid patients. Given the broad metabolic functions of triiodothyronine, its decrease can affect multiple organs and/or biological processes. For example, triiodothyronine enhances gonadotropin, LH, and FSH secretion by suppressing mRNA expression of the hypothalamic gonadotropin-inhibitory hormone [114]. Thus, a decrease in triiodothyronine level can lead to hypogonadism due to insufficient LH and/or FSH levels, as shown by a study with male rats [115]. We speculate that the decline in triiodothyronine level in COVID-19 can contribute to male hypogonadism.

#### Activins, inhibins, and follistatin

Activins A and B are members of the transforming growth factor-β superfamily of cytokines. They were initially recognized for their ability to release FSH from the anterior pituitary [116, 117]. Activin A was subsequently found to also stimulate GnRH in the rat hypothalamus [118] and in a murine GnRH-secreting neuronal cell line [119]. Its bioactivity is tightly regulated by two physiological inhibitors: inhibins (A and B) and follistatin, thereby forming the activin/inhibin/follistatin axis [120]. This metabolic axis is involved in a variety of biological processes, including inflammation and glucose metabolism [121, 122]. Serum levels of activin A, activin B, and follistatin are markedly increased in COVID-19 patients; the levels corelate with disease severity and in-hospital mortality [123, 124]. Since activin A promotes secretion of insulin from cultured pancreatic islet cells [125] and since elevated follistatin expression markedly increases insulin sensitivity in mouse skeletal muscle in vivo [126], it is possible that activation of the activin/inhibin/follistatin axis improves insulin responsiveness and glucose metabolism in COVID-19 patients. However, animal studies indicate that a dysregulated activin/inhibin/follistatin axis can damage the structure and immune environment in the testis and epididymis [127]. Thus, inflammation and diabetic complications in COVID-19 that disrupt the balance between activins and their antagonists, which is required for maintaining normal male reproductive organ function, can consequently affect male fertility by causing abnormal FSH and LH secretion via the hypothalamic–pituitary–testis axis (HPT) (Fig. 2).

## Concluding remarks

We are constantly reminded that COVID-19 is a public health crisis that threatens nearly all aspects of human life, with male patients being more likely than females to suffer from health complications. Given the unprecedented magnitude of the pandemic, its impact on male fertility could be substantial and could have long-lasting geopolitical effects. Inflammation and diabetes are among common co-morbidities of COVID-19. Given their roles as risk factors for male hypogonadism, the interactive detrimental impact of inflammation and diabetes on the reproductive organs of males could be greatly enhanced during COVID-19. Since the infection rate in young adults is increasing [128], we are concerned that the negative effect on male fertility may occur without severe respiratory symptoms, perhaps several months after acute infection (such has been reported for mumps virus-induced orchitis [129]). With SARS-CoV-2, which likely causes many undetected infections with subclinical organ abnormalities [130], even a moderate impact on male fertility could have profound population-based effects. To date, clinical studies have not focused on reproductive-age men with mild COVID-19 symptoms, plus no study has examined whether SARS-COV-2 infection further compounds male fertility in diabetic animals; thus, we cannot assess the quantitative importance of virus-mediated sub-fertility. However, the effect of COVID-19 complications, in particular inflammation and diabetes, on male fertility should enter prudent considerations of whether to vaccinate.

## Data Availability

Not applicable.

## Abbreviations

ACE2: Angiotensin-converting enzyme 2
CCL2: Chemokine (C–C motif) ligand 2
CD147: Cluster of differentiation 147
COVID-19: Coronavirus disease 2019
FSH: Follicle-stimulating hormone
GnRH: Gonadotropin-releasing hormone
HPT: Hypothalamic-pituitary-testis
IL: Interleukin
LH: Luteinizing hormone
ROS: Reactive oxygen species
SARS-CoV: Severe acute respiratory syndrome–related coronavirus
TNF: Tumor necrosis factor

## References

[1] Guan W-J, Ni Z-Y, Hu Y, Liang W-H, Ou C-Q, He J-X (2020). Clinical characteristics of coronavirus disease 2019 in China. N Engl J Med.

[2] Zhu N, Zhang D, Wang W, Li X, Yang B, Song J (2020). A Novel coronavirus from patients with pneumonia in China, 2019. N Engl J Med.

[3] Song E, Zhang C, Israelow B, Lu-Culligan A, Prado AV, Skriabine S (2021). Neuroinvasion of SARS-CoV-2 in human and mouse brain. J Exp Med.

[4] Puelles VG, Lütgehetmann M, Lindenmeyer MT, Sperhake JP, Wong MN, Allweiss L (2020). Multiorgan and renal tropism of SARS-CoV-2. N Engl J Med.

[5] Müller JA, Groß R, Conzelmann C, Krüger J, Merle U, Steinhart J (2021). SARS-CoV-2 infects and replicates in cells of the human endocrine and exocrine pancreas. Nat Metab.

[6] Ma MT, Badeti S, Chen C-H, Kim J, Choudhary A, Honnen B (2021). CAR-NK cells effectively target SARS-CoV-2-spike-expressing cell lines in vitro. Front Immunol.

[7] Yelin D, Wirtheim E, Vetter P, Kalil AC, Bruchfeld J, Runold M (2020). Long-term consequences of COVID-19: research needs. Lancet Infect Dis.

[8] Holmes E, Wist J, Masuda R, Lodge S, Nitschke P, Kimhofer T (2021). Incomplete systemic recovery and metabolic phenoreversion in post-acute-phase nonhospitalized COVID-19 patients: implications for assessment of post-acute COVID-19 syndrome. J Proteome Res.

[9] Haghpanah A, Masjedi F, Alborzi S, Hosseinpour A, Dehghani A, Malekmakan L (2021). Potential mechanisms of SARS-CoV-2 action on male gonadal function and fertility: current status and future prospects. Andrologia..

[10] Safarinejad MR (2008). Evaluation of endocrine profile, hypothalamic-pituitary-testis axis and semen quality in multiple sclerosis. J Neuroendocrinol.

[11] Maresch CC, Stute DC, Alves MG, Oliveira PF, de Kretser DM, Linn T (2018). Diabetes-induced hyperglycemia impairs male reproductive function: a systematic review. Hum Reprod Update.

[12] Abdel-Moneim A (2021). COVID-19 pandemic and male fertility: clinical manifestations and pathogenic mechanisms. Biochemistry.

[13] Boddu SK, Aurangabadkar G, Kuchay MS (2020). New onset diabetes, type 1 diabetes and COVID-19. Diabetes Metab Syndr.

[14] Stassen L, Armitage CW, van der Heide DJ, Beagley KW, Frentiu FD (2018). Zika virus in the male reproductive tract. Viruses.

[15] Govero J, Esakky P, Scheaffer SM, Fernandez E, Drury A, Platt DJ (2016). Zika virus infection damages the testes in mice. Nature.

[16] Ma W, Li S, Ma S, Jia L, Zhang F, Zhang Y (2016). Zika virus causes testis damage and leads to male infertility in mice. Cell.

[17] Hviid A, Rubin S, Mühlemann K (2008). Mumps. Lancet.

[18] Muciaccia B, Corallini S, Vicini E, Padula F, Gandini L, Liuzzi G (2007). HIV-1 viral DNA is present in ejaculated abnormal spermatozoa of seropositive subjects. Hum Reprod.

[19] Shevchuk MM, Nuovo GJ, Khalife G (1998). HIV in testis: quantitative histology and HIV localization in germ cells. J Reprod Immunol.

[20] Poretsky L, Can S, Zumoff B (1995). Testicular dysfunction in human immunodeficiency virus-infected men. Metabolism.

[21] Pudney J, Anderson D (1991). Orchitis and human immunodeficiency virus type 1 infected cells in reproductive tissues from men with the acquired immune deficiency syndrome. Am J Pathol.

[22] De Paepe ME, Waxman M (1989). Testicular atrophy in AIDS: a study of 57 autopsy cases. Hum Pathol.

[23] Hofny ER, Ali ME, Taha EA, Nafeh HM, Sayed DS, Abdel-Azeem HG (2011). Semen and hormonal parameters in men with chronic hepatitis C infection. Fertil Steril.

[24] Lyu Z, Feng X, Li N, Zhao W, Wei L, Chen Y (2017). Human papillomavirus in semen and the risk for male infertility: a systematic review and meta-analysis. BMC Infect Dis.

[25] Hoffmann M, Kleine-Weber H, Schroeder S, Krüger N, Herrler T, Erichsen S (2020). SARS-CoV-2 cell entry depends on ACE2 and TMPRSS2 and is blocked by a clinically proven protease inhibitor. Cell.

[26] Fagerberg L, Hallström BM, Oksvold P, Kampf C, Djureinovic D, Odeberg J (2014). Analysis of the human tissue-specific expression by genome-wide integration of transcriptomics and antibody-based proteomics. Mol Cell Proteomics.

[27] Wang Z, Xu X (2020). scRNA-seq profiling of human testes reveals the presence of the ACE2 receptor, a target for SARS-CoV-2 infection in spermatogonia leydig and sertoli cells. Cells.

[28] Pushkarsky T, Zybarth G, Dubrovsky L, Yurchenko V, Tang H, Guo H (2001). CD147 facilitates HIV-1 infection by interacting with virus-associated cyclophilin A. Proc Natl Acad Sci USA.

[29] Chen Z, Mi L, Xu J, Yu J, Wang X, Jiang J (2005). Function of HAb18G/CD147 in invasion of host cells by severe acute respiratory syndrome coronavirus. J Infect Dis.

[30] Wang K, Chen W, Zhang Z, Deng Y, Lian JQ, Du P (2020). CD147-spike protein is a novel route for SARS-CoV-2 infection to host cells. Signal Transduct Target Ther.

[31] Asgari R, Mansouri K, Bakhtiari M, Vaisi-Raygani A (2019). CD147 as an apoptosis regulator in spermatogenesis: deciphering its association with matrix metalloproteinases' pathway. Mol Biol Rep.

[32] Achua JK, Chu KY, Ibrahim E, Khodamoradi K, Delma KS, Iakymenko OA (2021). Histopathology and ultrastructural findings of fatal COVID-19 infections on testis. World J Mens Health.

[33] Chen L, Huang X, Yi Z, Deng Q, Jiang N, Feng C (2020). Ultrasound imaging findings of acute testicular infection in patients with coronavirus disease 2019: a single-center-based study in Wuhan, China. J Ultrasound Med.

[34] Flaifel A, Guzzetta M, Occidental M, Najari BB, Melamed J, Thomas KM (2021). Testicular changes associated with severe acute respiratory syndrome coronavirus 2 (SARS-CoV-2). Arch Pathol Lab Med.

[35] Li H, Xiao X, Zhang J, Zafar MI, Wu C, Long Y (2020). Impaired spermatogenesis in COVID-19 patients. EClinicalMedicine.

[36] Ma L, Xie W, Li D, Shi L, Ye G, Mao Y (2021). Evaluation of sex-related hormones and semen characteristics in reproductive-aged male COVID-19 patients. J Med Virol.

[37] Salonia A, Pontillo M, Capogrosso P, Gregori S, Tassara M, Boeri L (2021). Severely low testosterone in males with COVID-19: a case-control study. Andrology.

[38] Schuppe H-C, Meinhardt A, Allam JP, Bergmann M, Weidner W, Haidl G (2008). Chronic orchitis: a neglected cause of male infertility?. Andrologia.

[39] Gruschwitz MS, Brezinschek R, Brezinschek HP (1996). Cytokine levels in the seminal plasma of infertile males. J Androl.

[40] Loveland KL, Klein B, Pueschl D, Indumathy S, Bergmann M, Loveland BE (2017). Cytokines in male fertility and reproductive pathologies: immunoregulation and beyond. Front Endocrinol.

[41] Lei T, Moos S, Klug J, Aslani F, Bhushan S, Wahle E (2018). Galectin-1 enhances TNFα-induced inflammatory responses in Sertoli cells through activation of MAPK signalling. Sci Rep.

[42] Jiang Q, Maresch CC, Petry SF, Paradowska-Dogan A, Bhushan S, Chang Y (2020). Elevated CCL2 causes Leydig cell malfunction in metabolic syndrome. JCI Insight.

[43] Lainez NM, Coss D (2019). Leukemia inhibitory factor represses GnRH gene expression via cFOS during inflammation in male mice. Neuroendocrinology.

[44] Haedo MR, Gerez J, Fuertes M, Giacomini D, Páez-Pereda M, Labeur M (2009). Regulation of pituitary function by cytokines. Horm Res.

[45] Mehta P, McAuley DF, Brown M, Sanchez E, Tattersall RS, Manson JJ (2020). COVID-19: consider cytokine storm syndromes and immunosuppression. Lancet.

[46] Fijak M, Pilatz A, Hedger MP, Nicolas N, Bhushan S, Michel V (2018). Infectious, inflammatory and 'autoimmune' male factor infertility: how do rodent models inform clinical practice?. Hum Reprod Update.

[47] Yang M, Chen S, Huang B, Zhong JM, Su H, Chen YJ (2020). Pathological findings in the testes of COVID-19 patients: clinical implications. Eur Urol Focus.

[48] Rastrelli G, Di Stasi V, Inglese F, Beccaria M, Garuti M, Di Costanzo D (2021). Low testosterone levels predict clinical adverse outcomes in SARS-CoV-2 pneumonia patients. Andrology.

[49] Gacci M, Coppi M, Baldi E, Sebastianelli A, Zaccaro C, Morselli S (2021). Semen impairment and occurrence of SARS-CoV-2 virus in semen after recovery from COVID-19. Hum Reprod.

[50] Mannur S, Jabeen T, Khader MA, Rao LSS (2021). Post-COVID-19-associated decline in long-term male fertility and embryo quality during assisted reproductive technology. QJM.

[51] Montefusco L, Ben Nasr M, D'Addio F, Loretelli C, Rossi A, Pastore I (2021). Acute and long-term disruption of glycometabolic control after SARS-CoV-2 infection. Nat Metab.

[52] Chen S, de Craen AJ, Raz Y, Derhovanessian E, Vossen AC, Westendorp RG (2012). Cytomegalovirus seropositivity is associated with glucose regulation in the oldest old. Results from the Leiden 85-plus Study. Immun Ageing.

[53] Yoon JW, Ihm SH, Kim KW (1989). Viruses as a triggering factor of type 1 diabetes and genetic markers related to the susceptibility to the virus-associated diabetes. Diabetes Res Clin Pract.

[54] Roberts BW, Cech I (2005). Association of type 2 diabetes mellitus and seroprevalence for cytomegalovirus. South Med J.

[55] Ashfaq UA, Khalid H (2017). Mechanism of hepatitis C virus-induced diabetes mellitus. Crit Rev Eukaryot Gene Expr.

[56] Yang JK, Lin SS, Ji XJ, Guo LM (2010). Binding of SARS coronavirus to its receptor damages islets and causes acute diabetes. Acta Diabetol.

[57] Richardson S, Hirsch JS, Narasimhan M, Crawford JM, McGinn T, Davidson KW (2020). Presenting characteristics, comorbidities, and outcomes among 5700 patients hospitalized with COVID-19 in the New York City Area. JAMA.

[58] Nandy K, Salunke A, Pathak SK, Pandey A, Doctor C, Puj K (2020). Coronavirus disease (COVID-19): a systematic review and meta-analysis to evaluate the impact of various comorbidities on serious events. Diabetes Metab Syndr.

[59] Wang W, Shen M, Tao Y, Fairley CK, Zhong Q, Li Z (2021). Elevated glucose level leads to rapid COVID-19 progression and high fatality. BMC Pulm Med.

[60] Ren H, Yang Y, Wang F, Yan Y, Shi X, Dong K (2020). Association of the insulin resistance marker TyG index with the severity and mortality of COVID-19. Cardiovasc Diabetol.

[61] Codo AC, Davanzo GG, Monteiro LB, de Souza GF, Muraro SP, Virgilio-da-Silva JV (2020). Elevated glucose levels favor SARS-CoV-2 infection and monocyte response through a HIF-1α/glycolysis-dependent axis. Cell Metab.

[62] Zhu L, She ZG, Cheng X, Qin JJ, Zhang XJ, Cai J (2020). Association of blood glucose control and outcomes in patients with COVID-19 and pre-existing type 2 diabetes. Cell Metab.

[63] Mallapaty S (2020). Mounting clues suggest the coronavirus might trigger diabetes. Nature.

[64] Rubino F, Amiel SA, Zimmet P, Alberti G, Bornstein S, Eckel RH (2020). New-onset diabetes in Covid-19. N Engl J Med.

[65] Singh AK, Singh R (2020). Hyperglycemia without diabetes and new-onset diabetes are both associated with poorer outcomes in COVID-19. Diabetes Res Clin Pract.

[66] Liu F, Long X, Zhang B, Zhang W, Chen X, Zhang Z (2020). ACE2 expression in pancreas may cause pancreatic damage after SARS-CoV-2 infection. Clin Gastroenterol Hepatol.

[67] Steenblock C, Richter S, Berger I, Barovic M, Schmid J, Schubert U (2021). Viral infiltration of pancreatic islets in patients with COVID-19. Nat Commun.

[68] Laforge M, Elbim C, Frère C, Hémadi M, Massaad C, Nuss P (2020). Tissue damage from neutrophil-induced oxidative stress in COVID-19. Nat Rev Immunol.

[69] Yuan M, Konstantopoulos N, Lee J, Hansen L, Li Z-W, Karin M (2001). Reversal of obesity- and diet-induced insulin resistance with salicylates or targeted disruption of IKKbeta. Science.

[70] Houstis N, Rosen ED, Lander ES (2006). Reactive oxygen species have a causal role in multiple forms of insulin resistance. Nature.

[71] Gianchandani R, Esfandiari NH, Ang L, Iyengar J, Knotts S, Choksi P (2020). Managing hyperglycemia in the COVID-19 inflammatory storm. Diabetes.

[72] Michalakis K, Ilias I (2021). COVID-19 and hyperglycemia/diabetes. World J Diabetes.

[73] Hasan MM, El-Shal AS, Mackawy AMH, Ibrahim EM, Abdelghany E, Saeed AA (2020). Ameliorative effect of combined low dose of Pioglitazone and omega-3 on spermatogenesis and steroidogenesis in diabetic rats. J Cell Biochem.

[74] Maresch CC, Stute DC, Ludlow H, Hammes HP, de Kretser DM, Hedger MP (2017). Hyperglycemia is associated with reduced testicular function and activin dysregulation in the Ins2(Akita+/−) mouse model of type 1 diabetes. Mol Cell Endocrinol.

[75] Khalil ASM, Giribabu N, Yelumalai S, Shahzad H, Kilari EK, Salleh N (2021). Myristic acid defends against testicular oxidative stress, inflammation, apoptosis: restoration of spermatogenesis, steroidogenesis in diabetic rats. Life Sci.

[76] Simas JN, Mendes TB, Fischer LW, Vendramini V, Miraglia SM (2021). Resveratrol improves sperm DNA quality and reproductive capacity in type 1 diabetes. Andrology.

[77] Bosman E, Esterhuizen AD, Rodrigues FA, Becker PJ, Hoffmann WA (2015). Effect of metformin therapy and dietary supplements on semen parameters in hyperinsulinaemic males. Andrologia.

[78] Abu Bakar U, Subramaniam P, Kamar Bashah NA, Kamalrudin A, Kamaruzaman KA, Jasamai M (2020). Sperm proteomics analysis of diabetic induced male rats as influenced by *Ficus carica* leaf extract. Processes.

[79] Maresch CC, Stute DC, Fleming T, Lin J, Hammes HP, Linn T (2019). Hyperglycemia induces spermatogenic disruption via major pathways of diabetes pathogenesis. Sci Rep.

[80] Donath MY, Shoelson SE (2011). Type 2 diabetes as an inflammatory disease. Nat Rev Immunol.

[81] Esposito K, Nappo F, Marfella R, Giugliano G, Giugliano F, Ciotola M (2002). Inflammatory cytokine concentrations are acutely increased by hyperglycemia in humans. Circulation.

[82] Koenen TB, Stienstra R, van Tits LJ, de Graaf J, Stalenhoef AFH, Joosten LAB (2011). Hyperglycemia activates caspase-1 and TXNIP-mediated IL-1beta transcription in human adipose tissue. Diabetes.

[83] Dregan A, Charlton J, Chowienczyk P, Gulliford MC (2014). Chronic inflammatory disorders and risk of type 2 diabetes mellitus, coronary heart disease, and stroke: a population-based cohort study. Circulation.

[84] Aguirre V, Uchida T, Yenush L, Davis R, White MF (2000). The c-Jun NH(2)-terminal kinase promotes insulin resistance during association with insulin receptor substrate-1 and phosphorylation of Ser(307). J Biol Chem.

[85] Page ST, Amory JK, Bremner WJ (2008). Advances in male contraception. Endocr Rev.

[86] Ehses JA, Lacraz G, Giroix MH, Schmidlin F, Coulaud J, Kassis N (2009). IL-1 antagonism reduces hyperglycemia and tissue inflammation in the type 2 diabetic GK rat. Proc Natl Acad Sci USA.

[87] Kurauti MA, Costa-Júnior JM, Ferreira SM, Santos GJ, Sponton CHG, Carneiro EM (2017). Interleukin-6 increases the expression and activity of insulin-degrading enzyme. Sci Rep.

[88] Chen Y, Wang J, Liu C, Su L, Zhang D, Fan J (2020). IP-10 and MCP-1 as biomarkers associated with disease severity of COVID-19. Mol Med.

[89] Kleinert M, Clemmensen C, Hofmann SM, Moore MC, Renner S, Woods SC (2018). Animal models of obesity and diabetes mellitus. Nat Rev Endocrinol.

[90] Liu CY, Chang TC, Lin SH, Wu ST, Cha TL, Tsao CW (2020). Metformin ameliorates testicular function and spermatogenesis in male mice with high-fat and high-cholesterol diet-induced obesity. Nutrients.

[91] Nna VU, Bakar ABA, Ahmad A, Mohamed M (2019). Down-regulation of steroidogenesis-related genes and its accompanying fertility decline in streptozotocin-induced diabetic male rats: ameliorative effect of metformin. Andrology.

[92] Ye J, Luo D, Xu X, Sun M, Su X, Tian Z (2019). Metformin improves fertility in obese males by alleviating oxidative stress-induced blood-testis barrier damage. Oxid Med Cell Longev.

[93] Le H (2020). Metformin and systemic metabolism. Trends Pharmacol Sci.

[94] Tseng CH (2022). The effect of metformin on male reproductive function and prostate: an updated review. World J Mens Health.

[95] Yang D, Elner SG, Bian Z-M, Till GO, Petty HR, Elner VM (2007). Pro-inflammatory cytokines increase reactive oxygen species through mitochondria and NADPH oxidase in cultured RPE cells. Exp Eye Res.

[96] Sverrisson K, Axelsson J, Rippe A, Asgeirsson D, Rippe B (2015). Acute reactive oxygen species (ROS)-dependent effects of IL-1β, TNF-α, and IL-6 on the glomerular filtration barrier (GFB) in vivo. Am J Physiol Renal Physiol.

[97] Volpe CMO, Villar-Delfino PH, Dos Anjos PMF, Nogueira-Machado JA (2018). Cellular death, reactive oxygen species (ROS) and diabetic complications. Cell Death Dis.

[98] Petry SF, Sharifpanah F, Sauer H, Linn T (2017). Differential expression of islet glutaredoxin 1 and 5 with high reactive oxygen species production in a mouse model of diabesity. PLoS ONE.

[99] Bisht S, Faiq M, Tolahunase M, Dada R (2017). Oxidative stress and male infertility. Nat Rev Urol.

[100] Barroso G, Morshedi M, Oehninger S (2000). Analysis of DNA fragmentation, plasma membrane translocation of phosphatidylserine and oxidative stress in human spermatozoa. Hum Reprod.

[101] Zhang D-C, Chen R, Cai Y-H, Wang J-J, Yin C, Zou K (2020). Hyperactive reactive oxygen species impair function of porcine Sertoli cells via suppression of surface protein ITGB1 and connexin-43. Zool Res.

[102] Chernyak BV, Popova EN, Prikhodko AS, Grebenchikov OA, Zinovkina LA, Zinovkin RA (2020). COVID-19 and oxidative stress. Biochemistry (Mosc).

[103] Thau L, Gandhi J, Sharma S, Abai Babak (2021). Physiology, cortisol. StatPearls.

[104] Tan T, Khoo B, Mills EG, Phylactou M, Patel B, Eng PC (2020). Association between high serum total cortisol concentrations and mortality from COVID-19. Lancet Diabetes Endocrinol.

[105] Kobori Y, Koh E, Sugimoto K, Izumi K, Narimoto K, Maeda Y (2009). The relationship of serum and salivary cortisol levels to male sexual dysfunction as measured by the international index of erectile function. Int J Impot Res.

[106] Ringstrom SJ, Schwartz NB (1985). Cortisol suppresses the LH, but not the FSH, response to gonadotropin-releasing hormone after orchidectomy. Endocrinology.

[107] Gao HB, Tong MH, Hu YQ, Guo QS, Ge R, Hardy MP (2002). Glucocorticoid induces apoptosis in rat leydig cells. Endocrinology.

[108] Maiden MJ, Torpy DJ (2019). Thyroid hormones in critical illness. Crit Care Clin.

[109] Utiger RD (1995). Altered thyroid function in nonthyroidal illness and surgery. To treat or not to treat?. N Engl J Med.

[110] Malik J, Malik A, Javaid M, Zahid T, Ishaq U, Shoaib M (2021). Thyroid function analysis in COVID-19: a retrospective study from a single center. PLoS ONE.

[111] Warner MH, Beckett GJ (2010). Mechanisms behind the non-thyroidal illness syndrome: an update. J Endocrinol.

[112] Perros P, McCrimmon RJ, Shaw G, Frier BM (1995). Frequency of thyroid dysfunction in diabetic patients: value of annual screening. Diabet Med.

[113] Gu Y, Li H, Bao X, Zhang Q, Liu L, Meng G (2016). The relationship between thyroid function and the prevalence of type 2 diabetes mellitus in euthyroid subjects. J Clin Endocrinol Metab.

[114] Kiyohara M, Son YL, Tsutsui K (2017). Involvement of gonadotropin-inhibitory hormone in pubertal disorders induced by thyroid status. Sci Rep.

[115] Romano RM, Bargi-Souza P, Brunetto EL, Goulart-Silva F, Salgado RM, Zorn TMT (2018). Triiodothyronine differentially modulates the LH and FSH synthesis and secretion in male rats. Endocrine.

[116] Vale W, Rivier J, Vaughan J, McClintock R, Corrigan A, Woo W (1986). Purification and characterization of an FSH releasing protein from porcine ovarian follicular fluid. Nature.

[117] Ling N, Ying S-Y, Ueno N, Shimasaki S, Esch F, Hotta M (1986). Pituitary FSH is released by a heterodimer of the β-subunits from the two forms of inhibin. Nature.

[118] Calogero AE, Burrello N, Ossino AM, Polosa P, D'Agata R (1998). Activin-A stimulates hypothalamic gonadotropin-releasing hormone release by the explanted male rat hypothalamus: interaction with inhibin and androgens. J Endocrinol.

[119] González-Manchón C, Bilezikjian LM, Corrigan AZ, Mellon PL, Vale W (1991). Activin-A modulates gonadotropin-releasing hormone secretion from a gonadotropin-releasing hormone-secreting neuronal cell line. Neuroendocrinology.

[120] Wijayarathna R, de Kretser DM (2016). Activins in reproductive biology and beyond. Hum Reprod Update.

[121] Jones KL, de Kretser DM, Patella S, Phillips DJ (2004). Activin A and follistatin in systemic inflammation. Mol Cell Endocrinol.

[122] Hashimoto O, Funaba M (2011). Activin in glucose metabolism. Vitam Horm.

[123] Synolaki E, Papadopoulos V, Divolis G, Tsahouridou O, Gavriilidis E, Loli G (2021). The Activin/follistatin axis is severely deregulated in COVID-19 and independently associated with in-hospital mortality. J Infect Dis.

[124] McAleavy M, Zhang Q, Xu J, Pan L, Wakai M, Ehmann PJ (2021). Activin A correlates with the worst outcomes in COVID-19 patients, and can be induced by cytokines via the IKK/NF-kappa B pathway. BioRxiv.

[125] Hashimoto O, Funaba M, Litwack G (2011). Chapter eleven—activin in glucose metabolism. Vitamins & hormones.

[126] Han X, Møller LLV, De Groote E, Bojsen-Møller KN, Davey J, Henríquez-Olguin C (2019). Mechanisms involved in follistatin-induced hypertrophy and increased insulin action in skeletal muscle. J Cachexia Sarcopenia Muscle.

[127] Nicolas N, Michel V, Bhushan S, Wahle E, Hayward S, Ludlow H (2017). Testicular activin and follistatin levels are elevated during the course of experimental autoimmune epididymo-orchitis in mice. Sci Rep.

[128] Drake TM, Riad AM, Fairfield CJ, Egan C, Knight SR, Pius R (2021). Characterisation of in-hospital complications associated with COVID-19 using the ISARIC WHO clinical characterisation protocol UK: a prospective, multicentre cohort study. Lancet.

[129] Adamopoulos DA, Lawrence DM, Vassilopoulos P, Contoyiannis PA, Swyer GI (1978). Pituitary-testicular interrelationships in mumps orchitis and other viral infections. Br Med J.

[130] Oran DP, Topol EJ (2020). Prevalence of asymptomatic SARS-CoV-2 infection : a narrative review. Ann Intern Med.

